# The Association between COMT, BDNF, and NRG1 and Premorbid Social Functioning in Patients with Psychosis, Their Relatives, and Controls

**DOI:** 10.6064/2012/560514

**Published:** 2012-05-21

**Authors:** Muriel Walshe, Evangelos Vassos, Marco Picchioni, Madiha Shaikh, Timothea Toulopoulou, David Collier, Colm McDonald, Robin Murray, Elvira Bramon

**Affiliations:** ^1^NIHR Biomedical Research Centre for Mental Health, South London and Maudsley NHS Foundation Trust and Institute of Psychiatry, Kings College London, P.O. Box 63, De Crespigny Park, London SE5 8AF, UK; ^2^Department of Psychiatry, Clinical Science Institute, National University of Ireland, Galway, Ireland

## Abstract

We investigated the influences of putative candidate genes for psychosis on premorbid social adjustment and on premorbid schizoid-schizotypal traits. A family-based sample was used including 177 patients with schizophrenia or bipolar I disorder with a history of psychotic symptoms, 86 of their unaffected relatives, and 116 unrelated healthy controls. Association analyses on the combined sample were conducted using the Statistical Analysis for Genetic Epidemiology software (SAGE) and adjusting for age, sex, clinical group, and the family-based nature of the data. The COMT Val^158^Met and BDNF Val^66^Met polymorphisms showed no evidence of association with either phenotype. The SNP rs221533 of the NRG1 gene was significantly associated with premorbid adjustment in adolescence with TT homozygous subjects having a poorer performance than C allele carriers. In the context of neurodevelopmental disorders such as schizophrenia and other psychoses, this finding is plausible; however, it is preliminary and requires replication in an independent sample. In a broader sense, the use of intermediate quantitative phenotypes such as the ones presented in this study may be of help to understand the mechanism of action of genetic risk factors.

## 1. Introduction

Premorbid social dysfunction in patients with psychosis is well documented [1–3], but recent reporting of early social deficits in their unaffected relatives suggests that such impairments may be endophenotypic markers (indicators of shared genetic risk) for the disorder [4–6]. Catechol-o-methyl transferase (COMT), brain-derived neurotrophic factor (BDNF), and neuregulin 1 (NRG1) have all been implicated in the pathophysiology of schizophrenia [7–9] and bipolar disorder (BD) [7, 10]. There is also evidence supporting the association of the following single-nucleotide polymorphisms (SNPs) with risk for both disorders: COMT Val^158^Met [11, 12]; BDNF Val^66^Met [13, 14]; NRG1 rs221533 [15]. Investigating quantitative phenotypes that characterise psychotic disorders could increase the power to identify susceptibility genes and help to understand their mechanism of action [16, 17]. While the role of these particular SNPs has been investigated in neuroimaging [18–21], neurophysiology [22–24], and cognition endophenotypes [25–28], no study to date has tested their potential effects on early social function and personality traits.

We tested whether premorbid social functioning (PSF) and personality development in childhood and adolescence are impacted upon by variation within SNPs from three genes previously reported for their association with psychosis: NRG1 rs221533, COMT Val^158^Met, and BDNF Val^66^Met in patients with psychosis, their unaffected relatives, and community controls.

## 2. Materials and Methods

### 2.1. Description of Sample

The sample included 379 Caucasian subjects recruited from the Maudsley Family Psychosis Study (M : F = 132 : 82; mean age 33, sd 7.75) and the Maudsley Twin Study (M : F = 75 : 90; mean age 35, sd 10.71) whose mothers were interviewed about their social development using the premorbid adjustment scale (PAS) and premorbid schizoid-schizotypal Traits (PSST) scale [4, 29]. A total of 177 patients fulfilled DSM-III-R or DSM-IV diagnostic criteria for a psychotic disorder, including schizophrenia and bipolar disorder with a history of psychotic symptoms [30]. Unaffected first-degree relatives (*n* = 86) were free from psychotic illness (other psychiatric disorders were not an exclusion factor). Controls (*n* = 116) were included if they did not have a personal or family history of psychosis. A history of alcohol or substance dependence in the last 12 months, neurological diseases, and severe head injury were exclusion criteria for all groups. All participants gave their written informed consent. The study was approved by the Joint Ethical Committee for Research at the Institute of Psychiatry and South London and Maudsley NHS Foundation Trust.  Table 1 summarises the demographic characteristics of this sample.

### 2.2. PSA, PSST, and Clinical Scales

All participants (including controls) underwent a structured clinical interview with either the Schedule for Affective Disorders and Schizophrenia (SADS) or the Structured Clinical Interview for DSM Disorders (SCID) [30, 31] to allow for a DSM-IV diagnosis to be ascertained or ruled out. In addition, wherever available, clinical records were consulted for the patients. In order for the sample to be representative of the general population, nonpsychotic disorders such as a history of depression, anxiety, and other axis I disorders did not constitute exclusion criteria for any participant group. The unadjusted means and standard deviations for PSA and PSST scores are presented in Table 1.

A modified premorbid adjustment scale (PAS) was used to examine childhood and adolescent premorbid social function and personality development [32, 33]. The PAS scale assessed five areas of functioning (socialization, peer relations, academic achievement, school adaptation, andhobbies) over two consecutive time periods: 5–11 years(childhood—PAS1) and 12–16 years (adolescence—PAS2) [5]. Premorbid behavior and personality (age 12–16 years) were assessed using the premorbid schizoid and schizotypal traits (PSST) scale [34]. For both scales, higher scores denoted increased abnormality.

### 2.3. Genotype Analysis

Genomic DNA was extracted from saliva and blood samples using established procedures. The genotyping was performed in house on an ABI 7900 HT real-Time PCR system (Applied Biosystems) with standard Taqman allelic discrimination assays and under contract by KBiosciences (http://www.kbioscience.co.uk/). Quality control measures included placing three blank control samples on each plate and a minimum of four interplate and intraplate duplicated samples. Calling was performed using the SDS Software, and in cases of ambiguous calling, the genotyping was repeated. Genotype frequencies by clinical group are presented in Table 1.

### 2.4. Statistical Analysis

For the association of the premorbid scales with subsequent affection status (patients, relatives, controls), multivariate analysis was carried out with generalized estimating equations (GEEs) [35] with robust Hubert White sandwich estimators to accommodate intrafamilial correlations. Multiple linear GEE regression was used to compare log-transformed PAS and PSST scores of the patient and relative groups with the control group, controlling for age and gender. Statistical analyses were carried out in Stata v 10 [36] and SPSS v 15 [37]. All tests were two tailed using a 0.05 level of significance.

A separate analysis of the three groups (patients, relatives, and controls), although desirable, would lead to small, underpowered subsamples for an association study; therefore, we decided a priori to analyze the combined sample, controlling for age, gender, and affection status. Since the samples contained related individuals, tests of quantitative association of PAS and PSST with the three polymorphisms in pedigree data were conducted with the George-Elston regression method, which allows for familial (residual) association [38]. The primary association was performed employing an additive model of inheritance, and only significant findings were explored further with post hoc analyses under the dominant and recessive models. As the sample included monozygotic (MZ) twin pairs who have identical genotype, we randomly included only one twin from each MZ pair (60 MZ twins), while the dizygotic twins were treated as regular siblings. For this analysis, we utilized the ASSOC routine in the Statistical Analysis for Genetic Epidemiology software [39].

## 3. Results

### 3.1. PAS and PSST Group Comparisons

In a univariate analysis, there were statistically significant differences between the groups (patients, relatives, and controls) for PAS1 (*F* = 32.38, *df* = 2, *P* < 0.001), PAS2 (*F* = 33.94, *df* = 2, *P* < 0.001), and PSST (*F* = 22.91, *df* = 2, *P* < 0.001). In the multivariate linear regression analysis controlling for age and gender, patients had worse scores than controls on the PAS1 (*β* = 0.32, *P* < 0.001, 95%  CI = 0.23 − 0.40); PAS2 (*β* = 0.41, *P* < 0.001, 95%  CI = 0.30 − 0.52); PSST (*β* = 0.18, *P* < 0.001, 95%  CI = 0.12 − 0.24). The relatives group had worse scores than controls for PAS1 (*β* = 0.14, *P* = 0.001, 95%  CI = 0.05 − 0.23) and PAS2 (*β* = 0.19, *P* < 0.001, 95%  CI = 0.08 − 0.30) but not for PSST (*β* = 0.02, *P* = 0.38, 95%  CI = − 0.03 − 0.09).  Table 1 summarises the demographic characteristics of this sample, the PAS and PSST scores as well as genotype frequencies by clinical group.

### 3.2. Genetic Association Analysis

We conducted an association study of the combined sample, controlling for age, gender, and affection status. All three SNPs studied were in Hardy-Weinberg equilibrium (*P* > 0.10). We found a nominal association between variation in NRG1 and PAS2 (*P* = 0.017) but not with PAS1 or PSST (*P* > 0.10). Subjects with the TT genotype had worse premorbid functioning in adolescence. Post hoc analysis under the recessive model (TT versus C allele carriers) marginally increased the significance of this association (*P* = 0.0085). With the exception of a trend for the COMT val/val variant to be associated with higher PSST scores (*P* = 0.07), we found no other significant associations for BDNF or COMT. There was no significant association between the 3 SNPs and the presence of psychosis. Since the traits under study (PAS and PSST) are correlated, a multiple testing adjustment for the three loci investigated would suffice, and indeed, the association with NRG1 does remain after such adjustment.

## 4. Discussion

While impaired PSF has been shown to be associated with genetic risk for schizophrenia, only a few studies have attempted to investigate associations between PSF and genetic variation in candidate genes for psychosis [40, 41]. Goghari and Sponheim [42] found no link between scores on the PAS and COMT similar to our study. We failed to find an association between BDNF and PSF, but with no other published studies investigating this, no firm conclusions can be drawn here. COMT Val^158^Met and BDNF Val^66^Met polymorphisms have long been identified as candidate genes for schizophrenia, but recent meta-analyses and large-scale genome-wide association studies of schizophrenia [43–45] and bipolar disorder [46, 47] have failed to confirm their direct role in the etiology of these diseases. However, the effect of these two loci in cognition remains a strong and well replicated finding [48–52], which would make their role in social functioning and personality development plausible. Our negative findings, however, do not lend support to this hypothesis.

Our study has a number of limitations. Firstly, our sample, which includes both patients with schizophrenia and with bipolar disorder, was not large enough to subdivide it by disease, and thus, only a combined analysis could be undertaken. A heterogeneous sample like this (rather than a more specific disease focused one) could have reduced the power of the study to find associations between the candidate genes and the clinical phenotype. While our study was cross-sectional with retrospective assessments, to assess dynamic concepts like social function and personality, a prospective cohort approach would have been preferable.

Our preliminary findings suggest that NRG1 rs221533 could influence premorbid adjustment during adolescence, with the T allele being associated with poorer performance. This result is supported under the recessive model (TT versus TC and CC) and survives a multiple testing adjustment (for the three SNPs investigated). While the C allele was originally proposed as the risk allele for schizophrenia, no allelic variant of this SNP has been homogeneously implicated in subsequent replication studies [53]. For instance, the T allele was found to be overtransmitted in a Dutch schizophrenia sample [54] and in childhood onset schizophrenia [55] and to predict worse response to antipsychotics than the C allele [56]. In short, the role of NRG1 rs221533 in psychosis as well as in neurodevelopment is still not fully understood, and its expression may be influenced by other genetic, epigenetic, or environmental factors [53].

Finally, in this study, no significant association between the three SNPs selected and psychosis was observed. Of course our sample was clearly underpowered to test this kind of association due to the small effect size of common SNPs contributing to disease susceptibility [57–60]. The effect of NRG1 on a quantitative trait like PAS is probably stronger, hence the association observed in our study. Clearly, this is a preliminary study, and our finding that the T allele is associated with impaired social functioning in adolescence is exploratory and needs to be replicated in an independent sample. Nevertheless, we believe that intermediate phenotypes for psychotic disorders, like the ones we presented here as well as other measures of brain development and function, have the potential to help towards our understanding of the mechanisms by which genetic variation leads to the onset of the disease.

## Figures and Tables

**Table 1:** Demographics, scale scores, and genotype frequencies.

	Patients	Relatives	Controls	Total
Age (years)				
*N*	177	86	116	379
Mean	32.8	33.4	36.3	34.0
SD	7.9	8.5	11.0	9.2
Minimum	17.0	18.0	19.0	17.0
Maximum	55.0	56.0	62.2	62.2
Sex				
% females	30.5	47.7	66.4	45.4
PSA1 scores at 5–11 years				
*N*	168	83	101	352
Mean	11.7	9.5	8.0	10.1
SD	4.5	3.4	1.8	4.0
Minimum	5.5	6.0	3.0	3.0
Maximum	26.0	24.5	12.0	26.0
PSA2 scores at 12–16 years				
*N*	168	83	100	351
Mean	12.5	9.8	7.9	10.5
SD	5.4	4.0	2.5	4.8
Minimum	5.5	4.5	2.5	2.5
Maximum	30.0	25.5	17.0	30.0
Total PSST score				
*N*	175	85	113	373
Mean	11.5	9.7	9.2	10.4
SD	3.4	2.5	1.6	3.0
Minimum	8.0	8.0	8.0	8.0
Maximum	26.0	19.0	15.0	26.0
NRG1				
CC	15	10	10	35
TC	78	43	48	169
TT	65	24	52	141
COMT				
Met/Met	41	19	34	94
Val/Met	84	38	45	167
Val/Val	47	24	31	102
BDNF				
Met/Met	4	1	2	7
Val/Met	47	22	36	105
Val/Val	120	60	75	255
